# The efficacy of indwelling pleural catheter placement versus placement plus talc sclerosant in patients with malignant pleural effusions managed exclusively as outpatients (IPC-PLUS): study protocol for a randomised controlled trial

**DOI:** 10.1186/s13063-015-0563-y

**Published:** 2015-02-12

**Authors:** Rahul Bhatnagar, Brennan C Kahan, Anna J Morley, Emma K Keenan, Robert F Miller, Najib M Rahman, Nick A Maskell

**Affiliations:** Academic Respiratory Unit, University of Bristol, Southmead Hospital, Learning and Research Building, Southmead Road, Bristol, BS10 5NB UK; Respiratory Research, Clinical Research Centre, Southmead Hospital, Southmead Road, Bristol, BS10 5NB UK; Pragmatic Clinical Trials Unit, Queen Mary University of London, 58 Turner Street, London, E1 2AB UK; Research Department of Infection and Population Health, Institute of Epidemiology and Healthcare, University College London, 222 Euston Road, London, NW1 2DA UK; Clinical Research Department, London School of Hygiene and Tropical Medicine, Keppel Street, London, WC1E 7HT UK; Oxford Centre for Respiratory Medicine, Churchill Hospital, Old Road, Oxford, OX3 7LE UK; Oxford Respiratory Trials Unit, University of Oxford, Churchill Hospital, Old Road, Oxford, OX3 7LE UK

**Keywords:** Catheters, indwelling, Chest tubes, Outpatients, Pleural effusion, malignant, Pleurodesis, Sclerosing solutions, Talc, Randomised controlled trial

## Abstract

**Background:**

Malignant pleural effusions (MPEs) remain a common problem, with 40,000 new cases in the United Kingdom each year and up to 250,000 in the United States. Traditional management of MPE usually involves an inpatient stay with placement of a chest drain, followed by the instillation of a pleural sclerosing agent such as talc, which aims to minimise further fluid build-up. Despite a good success rate in studies, this approach can be expensive, time-consuming and inconvenient for patients. More recently, an alternative method has become available in the form of indwelling pleural catheters (IPCs), which can be inserted and managed in an outpatient setting. It is currently unknown whether combining talc pleurodesis with IPCs will provide improved pleural symphysis rates over those of IPCs alone.

**Methods/Design:**

IPC-PLUS is a patient-blind, multicentre randomised controlled trial (RCT) comparing the combination of talc with an IPC to the use of an IPC alone for inducing pleurodesis in MPEs. The primary outcome is successful pleurodesis at five weeks post-randomisation. This study will recruit 154 patients, with an interim analysis for efficacy after 100 patients, and aims to help to define the future gold standard for outpatient management of patients with symptomatic MPEs.

**Discussion:**

IPC-PLUS is the first RCT to examine the practicality and utility of talc administered via an IPC. The study remains in active recruitment and has the potential to significantly alter how patients requiring pleurodesis for MPE are approached in the future.

**Trial registration:**

This trial was registered with Current Controlled Trials (identifier: ISRCTN73255764) on 23 August 2012.

## Background

Malignant pleural effusions (MPEs) are a common complication of many cancers, with 40,000 new cases in the United Kingdom each year and up to 250,000 in the United States [1]. Their presence usually indicates metastatic disease, and hence possibly a poorer prognosis.

The traditional management of MPE involves inpatient insertion of a chest drain, to ensure fluid drainage and pleural apposition, before the instillation of a sclerosant substance to cause pleural inflammation and adhesion, also known as pleurodesis. Many substances can be used as a pleural irritant, although by far the most commonly used worldwide is talc, which has been shown to be superior to numerous alternatives [2].

Quoted pleurodesis success rates are typically high with talc, ranging from 81 to 100% [3], although these figures may vary considerably in real-world practice due to differences between clinicians and between the practices of individual centres. To achieve such high efficacy, a patient typically requires admission for the insertion of a chest tube and drainage. Only once the pleural space is felt to be dry is the talc inserted. This usually requires an inpatient stay of five to seven days [4,5], which can have a significant health economic impact, as well as the potential to impair the quality of remaining life for patients. Following the widespread use of large-particle talc, the side effects of pleurodesis have tended to be minor, the commonest of which are fever, pain and gastrointestinal upset [2,6,7], although there have been rare cases of empyema [8].

The main drawback of the traditional method of pleurodesis is the length of hospital stay and the inconvenience to patients. In more recent years, indwelling pleural catheters (IPCs) have become more widely used and may the potential to alleviate these problems.

IPCs are silastic tubes, which have the potential to be left in place for weeks to months after being tunnelled under the skin. They can be inserted under local anaesthetic or at thoracoscopy, and are usually performed as a day case. Once at home, the aim is to drain fluid regularly (usually three times per week) in the patient’s own environment. This maximises the opportunity for pleural apposition and adhesion, which potentially leads to complete pleurodesis. Drainage can be performed by anyone with appropriate training, including the patient, but is often managed by district nursing teams.

IPCs have been shown to be effective in the management of MPEs, although there is a paucity of evidence comparing them directly to talc pleurodesis. In a retrospective series of 250 cases, almost 90% of patients experienced complete or partial relief of dyspnoea [9], a finding bettered in a later study in which all patients experienced improvement [10]. Indwelling drains have also been shown to improve other outcomes, such as length of hospital stay and future admissions, even in comparison to talc pleurodesis [4,11]. Despite the need for proprietary drainage kits, they may also be cheaper overall to healthcare providers, if used for limited periods of time [12].

Regardless of patients’ short life expectancies, this is an achievable goal as IPCs can often be removed following sustained reduction in drainable fluid volumes, a reliable surrogate indicator for pleurodesis. Such spontaneous pleurodesis generally occurs in around 50% of cases [4,10,13] and is heavily influenced by the underlying tumour type [14], although rates as high as 70% were reported in one study [15]. The presence of ‘trapped lung’ (usually due to central airways obstruction or visceral pleural fibrosis) can lead to incomplete expansion following pleural fluid drainage, which no doubt influenced the variability of the time to pleurodesis in these studies. Indeed, in patients with trapped lung, the persistent failure of pleural apposition makes the achievement of any degree of pleurodesis much less likely overall, meaning regular fluid management with an IPC may be the only feasible approach to their care.

However, IPCs are not without drawbacks. There may be significant pain associated with the immediate and short-term post-procedure period, and in some cases pleural tract metastases have been documented [16]. Complications including empyema formation (3%), secondary fluid loculation (12%) and cellulitis (2%) have also been reported [9]. Nevertheless, meta-analysis data has shown IPCs are generally safe to use, with an overall complication rate of 12.5%.

It would seem, therefore, that the optimal approach to the management of MPEs should be the combination of talc instillation, to achieve the highest pleurodesis rates and long-term fluid prevention, and placement of an IPC to allow greater convenience and quality of life for the patient. This should also theoretically lead to reduced overall healthcare costs when compared to either individual method. Despite the potential for combining fluid management approached being recognised in the literature, [17] there have been no studies to date to test this hypothesis, although ambulatory pleurodesis for malignant effusions was attempted in one small series by Saffran *et al*. [18]. In this study, a closed-system pigtail catheter was inserted and pleurodesis was attempted at a later date using four grams of talc. Patients were managed as outpatients and the authors describe their method as being a viable alternative to traditional inpatient management. However, patient numbers were limited to 10 and there was no attempt at randomisation. The study took place before the widespread introduction of IPCs.

The IPC-PLUS trial aims to test the hypothesis that the combination of talc in addition to IPCs is superior to IPCs alone in the management of MPEs. This trial has the potential to significantly affect, on a global scale, the way in which such effusions are managed in the future.

## Methods/Design

### Study questions

Our primary research question is, ‘In patients with a proven MPE, does the use of talc as a sclerosant in conjunction with an IPC increase the number of patients achieving successful pleurodesis, when compared to using an IPC alone?’

Our secondary research questions are as follows:

1. Does using talc and an IPC together alter the amount of pain and breathlessness a patient experiences, when compared to using an IPC alone?
2. Does the use of talc and an IPC together alter a patient’s quality of life, when compared to using an IPC alone?
3. What are the medical complications of using talc in conjunction with an IPC?
4. What are the logistical and clinical difficulties with using talc in conjunction with an IPC?
5. Does the combination of talc and an IPC together influence the degree of fluid septation and loculation seen on thoracic ultrasound?
6. Does the baseline level of serum brain natriuretic peptide (BNP) correlate with the volume of pleural fluid drained and chance of successful pleurodesis?
7. Does pleural elastance during initial drainage correlate with lung entrapment and the chance of successful pleurodesis?
8. Is using talc in combination with IPC cost-effective when compared to IPC alone?

### Sample size and power calculation

Talc pleurodesis alone has been shown to be up to 90% efficacious in trial conditions [3], and we expect the combination of talc and IPC to be at least as effective as talc alone. IPCs used alone have a more variable range for pleurodesis efficacy, but suggest an average rate of around 50%.

Therefore, in order to detect a 25% difference in pleurodesis success at five weeks (using conservative estimates of 60% IPC alone versus 85% IPC and talc) with 90% power, a 5% significance level and 5% loss to follow-up, we would require 154 patients (77 in each arm). An interim analysis for efficacy will take place after 100 patients are randomised.

### Ethics, approvals and sponsorship

The study is sponsored in the United Kingdom by North Bristol NHS Trust, and has been granted the necessary (national) approvals by both the Oxford A Research Ethics Committee (approval number: 12/SC/0242) and the Medicines and Healthcare Products Regulatory Agency (MHRA) (EudraCT number: 2012-000599-40).

### Investigational product: Novatech Steritalc®

Medicinal sterile talc as used in this trial is mined in Luzenac, France. It is marketed in the United Kingdom as Steritalc® and imported by GB UK Healthcare Ltd (Selby, UK). Talc is a naturally occurring mineral which, when processed for medical use as Steritalc, takes the form of a white powder of controlled particle size (graded). It is not licensed in the United Kingdom but is commonly used for the induction of pleurodesis, usually to prevent recurrence of MPEs or pneumothoraces. Medicinal talc has been licensed by the Food and Drug Administration (FDA) in the United States since 2003. Prior to introduction into the pleural cavity it is reconstituted into slurry using an inert solvent such as 0.9% saline. The typical dose of talc is two to four grams. Common side effects following pleural administration of talc are mild pleuritic pain and low-grade fever.

### Study setting and design

The IPC-PLUS study is a non-commercial, patient-blind, multicentre, randomised controlled trial of a medicinal product. Patients will be recruited from multiple centres within the United Kingdom. The trial is supported by the appropriate local and regional cancer networks.

Clinical care, drain insertion and imaging will be provided by local medical professionals at the patients’ base hospitals or appropriate satellite centres. Further care will be provided by ward and specialist nurses in these centres, who will also be available for telephone support. Routine drainage of pleural fluid will take place in the community and at follow-up visits. All drainages up to the 28-day post-randomisation visit will be performed by appropriately trained medical staff such as district nurses, lung cancer specialist nurses or research nurses. After this, drainages may be performed by anyone who has been appropriately trained (except the patient themselves).

### Patient population

Trial patients will be recruited from those presenting with symptomatic MPEs. As part of their normal clinical care, it will have been decided that outpatient treatment with an IPC is the most appropriate strategy for fluid management.

### Inclusion criteria

The inclusion criteria for this trial are as follows:

1. Symptomatic MPE, agreed at appropriate local or regional level to require an IPC, defined as pleural fluid in the context of one of the following: a histocytologically proven pleural malignancy; an otherwise unexplained pleural effusion in the context of clinically proven cancer elsewhere or a radiologically proven pleural malignancy, as diagnosed in normal clinical practice on thoracic CT, in the absence of histocytological proof.
2. Expected survival of more than two months and the Eastern Co-operative Oncology Group/World Health Organisation (ECOG/WHO) performance status of two or more. Patients with a performance status of three may be included if it is felt that removal of the pleural fluid would improve their performance status to two or better.
3. Written informed consent to trial participation.

### Exclusion criteria

The exclusion criteria for this trial are as follows:

1. Aged under 18 years.
2. Females who are pregnant or lactating.
3. Patient is unable to provide informed consent.
4. Previous attempts at pleurodesis have been made within the last 56 days on the same side as the effusion requiring management.
5. Previously documented adverse reaction to talc or lidocaine.
6. Community services are unable to drain the IPC at least twice per week.
7. Evidence of extensive lung entrapment on a chest X-ray or computed tomography (CT) scan, or significant fluid loculation on an ultrasound scan, to a level which would normally be a contraindication to attempted talc pleurodesis or IPC insertion.
8. Other contraindication to IPC insertion.
9. Patient has no access to a telephone.

### Screening and consent

Patients will be screened using the inclusion and exclusion criteria as described above. Screening logs documenting reasons for exclusions will be kept throughout the trial. Those who may be suitable for an IPC will have this option discussed in a normal outpatient or inpatient setting, where they will also be given the option of participating in the IPC-PLUS trial. Eligible patients will be invited to participate on a consecutive basis, and will be provided with an information leaflet at the earliest opportunity. They will be allowed sufficient time, as determined by the patient, to fully consider trial entry, as well as to ask questions of investigators. Written informed consent to trial participation will be obtained prior to enrolment. Consent must be taken by a member of the trial team and should take place before the placement of the patient’s IPC.

### Trial interventions

**Figure 1:**
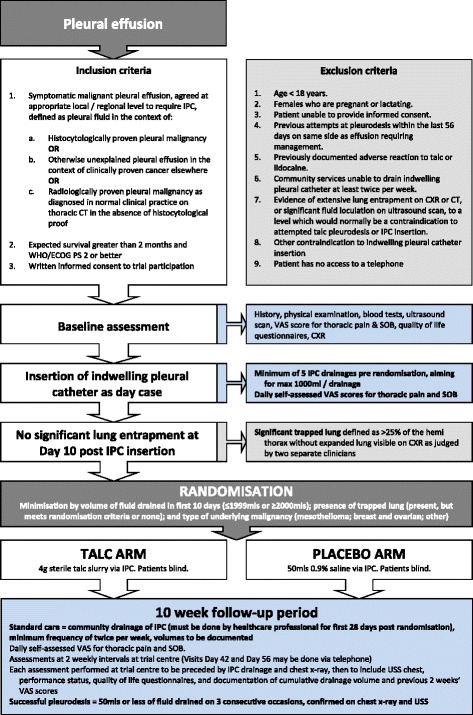
**Summary flow chart for IPC-PLUS trial.** IPC = Indwelling pleural catheter, WHO/ECOG = World Health Organisation/Eastern Cooperative Oncology Group, PS = Performance status, CXR = Chest X-ray, CT = Computed tomography, VAS = Visual analogue scale, SOB = Shortness of breath, USS = Ultrasound scan.

The trial interventions are summarised in Table 1 and in Figure 1 (trial flow chart).

**Table 1:** **Visit schedule**

	**Timings**										
**Event**	**Pre-screening**	**Consent/baseline**	**IPC insertion**	**Pre-randomisation**	**Randomisation**	**Follow-ups (days post-randomisation)**					**On-going**
						**14**	**28**	**42** ^**a**^	**56** ^**a**^	**70**	
Provide patient information sheet	X										
Sign consent		X									
Thoracic ultrasound		X			X	X	X	X	X	X	
Chest X-ray			X		X	X	X	X	X	X	
Standard blood tests		X									
Trial blood samples (Southmead and Oxford only)		X									
Trial pleural fluid samples (Southmead and Oxford only)			X		X	X	X	X	X	X	
Manometry			X								
Instillation of talc/placebo					X						
Community IPC drainages				X^b^							X
Drainage booklet			X								X
Daily VAS scores				X	X						X
Collection of VAS booklet					X	X	X	X	X	X	
EQ-5D questionnaire		X			X	X	X	X	X	X	
QLQ-C30 questionnaire		X			X	X	X	X	X	X	
Patient diary				X							X

IPC = Indwelling pleural catheter.

VAS = Visual analogue scale.

EQ-5D = EuroQuol 5D.

QLQ-C30 = Quality of Life Questionnaire C30.

^a^Visits at days 42 and 56 may be done over the telephone and therefore the patient would not have a chest X-ray or thoracic ultrasound.

^b^Minimum of three drainages in the community between IPC insertion and randomisation.

### Pre-randomisation

Following consent, a baseline assessment will be undertaken by a member of the trial team and entered onto the appropriate case report form (CRF). This will include:

1. Relevant medical history and physical examination, to include the onset and nature of symptoms, type of malignancy causing effusion (if known), pleural procedures to date, current ECOG/WHO performance status, current analgesia history and current and projected treatment plan outside of IPC-PLUS;
2. Results of standard blood tests (from within 24 hours);
3. Visual-Analogue Scale (VAS) score to assess thoracic pain and breathlessness;
4. Quality of life assessment using EuroQol 5D (EQ-5D) and Quality of Life Questionnaire C30 (QLQ-C30) health questionnaires;
5. Chest X-ray, ideally posterior-anterior (from within previous 10 days) and
6. Thoracic ultrasound scan.

Patients will then be given an appointment, if this has not already been provided, to have an IPC (PleurX® catheter, CareFusion, IL, USA) inserted as a day case procedure within one week of the baseline assessment.

IPCs must be placed by an appropriately trained member of staff, but not necessarily a member of the trial team. Immediately following drain placement, a therapeutic aspiration should be performed. During drainage, patients should have pleural pressures measured after every 100 to 200 mls of fluid removed, using a calibrated electronic pleural manometer (Mirador Biomedical, Seattle, WA, USA). Pressure measurements should be recorded along with the total volume removed. A chest X-ray should be performed post-procedure to confirm adequate drain placement.

Prior to discharge, the patient will be issued with a drainage booklet which will act as a record for the volumes of fluid drained throughout their period of trial participation. They will also be given a chart on which they can complete their own VAS scores for pain and breathlessness, which should be done on a daily basis.

For the period post IPC insertion and before their randomisation visit, patients should have their fluid drained on at least five occasions, the initial drainage being immediately after IPC insertion prior to discharge. This first drainage may be to the maximum clinically appropriate volume, with subsequent drainages to a maximum of 1,000 mls per drainage. The patient’s fifth drainage can take place as part of their randomisation visit.

Patients will attend their local trial centre 10 days (+/− one day, as above) after IPC insertion. Their pleural space should be drained to dryness, or as close to dryness as allowed by symptoms. Following this, they should undergo a chest X-ray (ideally posterior-anterior) and have an appointment with a member of the trial team, who will perform a standardised medical assessment. Quality of life will be assessed using the EQ-5D and QLQ-C30 questionnaires. The chest X-ray should be examined for evidence of lung entrapment and significant fluid. A thoracic ultrasound of the side where the IPC has been inserted should be performed, looking for evidence of fluid loculation and septation.

If there is evidence of significant lung entrapment (defined as >25% of the hemithorax without expanded lung visible on a chest X-ray, as judged by two separate clinicians) or significant pleural fluid (defined as pleural fluid, confirmed on thoracic ultrasound, occupying more than one third of the hemithorax as judged by two separate clinicians using visual estimation on a chest X-ray), then the patient should be excluded from randomisation. Patients who do not meet the criteria for randomisation should have their on-going care devolved to the appropriate local services. Patients may also be excluded for other clinical reasons not relating to the degree of lung entrapment or residual fluid. If a patient is eligible for trial continuation at this point then they should be randomised at the same visit and given the allocated treatment substance before returning home.

### Randomisation, blinding and emergency unblinding

Those who are eligible for will be randomly assigned in a 1:1 ratio to either receive intrapleural talc slurry (4 g Novatech Steritalc mixed with 50 mls 0.9% saline) via the IPC, or to receive a placebo instillation of 0.9% sterile saline alone.

Treatment allocation will be performed by an independent computer randomisation service, which will be accessed by the main trial coordination centre on behalf of recruitment centres following confirmation of suitability for randomisation. Minimisation with a random component will be used [19].

The minimisation factors are:

1. Volume of pleural fluid removed in the first 10 days post IPC (≤1,999 mls or ≥2,000 mls),
2. Malignancy subtype (ovarian and breast, mesothelioma or other), and
3. Day 10 chest X-ray appearance (expanded with no evidence of trapped lung or evidence of trapped lung but fits the criteria for randomisation).

The study is to be performed in a single blind fashion. Patients are to be kept unaware of their treatment allocation, but the physician and other healthcare professionals involved with administering the slurry or placebo are made aware of the allocation. A number of methods are to be used to reduce the likelihood of a patient learning of their allocation. These include:

1. Making the randomisation phone call in a separate room to the patient,
2. Preparing the slurry or placebo in a separate room to the patient and ensuring materials are covered before the patient is brought in,
3. Opaque syringes being used to make it less clear which substance is being administered and
4. The slurry or placebo being administered from behind the patient, with the patient facing forward.

Patients may have their treatment allocation revealed (unblinded) at any time according to clinical need. A 24-hour telephone number will be available for unblinding queries.

### Post-randomisation

The administration of the randomised substance should be followed by an adequate flush to ensure as little as possible is left in the IPC line. Patients should then be observed for a minimum of two hours before being discharged home. Observations should include at least half-hourly measurements of pulse, blood pressure, temperature, pain score and respiratory rate. Patients must have their first post-randomisation drainage between 12 and 36 hours after instillation, and early communication with community nursing teams is vital to ensure this takes place.

### Community drainage

Following randomisation, all patients should receive fluid drainage in the community, although if necessary patients may attend their local trial centre. Drainages will be done by an appropriately trained healthcare professional up to and including the day 28 follow-up visit. After this, until the end of the trial follow-up period, drainages may be performed by anyone with an appropriate level of training. This may include the patient’s family or carers, but should not be the patient themselves. The frequency of drainage will be at the discretion of the patient and community team, but should occur at least twice per week, and should begin at three times per week. Drainage volumes will be recorded on each occasion.

### Clinical assessments (days 14, 28, 42, 56 and 70 post-randomisation)

The follow-up period for each patient is 10 weeks post-randomisation, or until death. During this time, the first clinical assessment will occur 14 days after randomisation, and at two-weekly intervals thereafter. The appointments scheduled for days 42 and 56 may take place over the telephone. Appointments on days 14, 28 and 70 must take place at the base hospital or satellite centre.

### Face-to-face appointments (mandatory on days 14, 28 and 70, optional on days 42 and 56)

Before each assessment, but following arrival at the trial centre, the patient’s IPC should be drained to dryness by a trained member of staff. Patients should also have a chest X-ray (ideally posterior-anterior) after they are drained. The assessment should then be completed and will include:

1. A record of any contact with medical services including hospital admissions and length of stay, outpatient care visit, emergency care visit and ambulance service use Complications of IPC placement through history and examination;
2. Documentation of analgesia requirements (day 14 only);
3. Documentation of chemotherapy and/or radiotherapy and any response;
4. Current ECOG/WHO performance status;
5. Quality of life assessments using EQ-5D and QLQ-C30 health questionnaires and
6. A thoracic ultrasound scan, alongside completing the ultrasound CRF.

### Telephone appointments (optional on days 42 and 56)

Any appointment which is to be performed over the telephone should consist of the following:

1. A verbal reminder to the patient to complete and send their quality of life questionnaires and VAS booklets back to their local trial centre, ensuring that a VAS score is completed during the telephone consultation.
2. Completion of a specific telephone follow-up CRF by the researcher, along with the standard health service use CRF.
3. A review of drainage volumes with the patient over the telephone.

If drainage volumes appear to have reduced to a level suggesting pleurodesis, or if there is any suspicion of a drainage or IPC complication, then the patient must attend for the next scheduled follow-up visit. Alternatively, a patient may attend the following day for a full face-to-face visit, with the telephone follow-up being discarded.

### Removal of drains

Once inserted, drains may be removed at any time at the clinical discretion of the patient’s primary physician, at the request of the patient or at the discretion of the trial team. If a drain is to be removed, patients should be given an appointment to have this done within 14 days of the clinical assessment at which this decision was taken. Any patient who has a drain removed during their post-randomisation trial period will continue to undergo planned follow-up for the full 70 days.

### Blockage of drains

All care should be taken to ensure IPCs do not become blocked, beginning with an adequate flush at the end of sclerosant administration. If there is a suspicion that a blockage has occurred then standard local unblocking procedures should be followed.

### Biological samples and storage

During the trial baseline assessment, all patients should have standard blood tests for full blood count, urea and electrolytes, liver function, clotting function and C-reactive protein taken if there are no results available from within the previous 24 hours. In addition to these, at the research sites at North Bristol and Oxford, one ethylenediaminetetraacetic acid (EDTA), one serum gel tube and one citrate tube of blood should be taken. During IPC insertion, one EDTA, one serum gel tube and one citrate sample tube of pleural fluid should also be collected from patients at the North Bristol and Oxford sites. All such trial samples should be processed and stored as per the appropriate standard operating procedure.

At the North Bristol and Oxford research sites, prior to each trial follow-up appointment (every two weeks for 10 weeks), additional samples of pleural fluid should be collected during IPC drainage, before being processed and stored in the same manner as above.

Participants will give their permission for linked anonymous blood and pleural samples to be stored and analysed at North Bristol NHS Trust (NBT), or, if from another site, for those samples to be transferred to NBT for storage and analysis. Samples will be stored in a dedicated freezer in the University of Bristol laboratory on the NBT site. Samples will be stored, anonymised and eventually destroyed in line with local policy.

### Ultrasound scans

All ultrasound scans must be performed by fully trained operators (with sufficient experience to scan and interpret images independently) on the local research team. Scans will be used to assess the presence and degree of pleural fluid complexity, and fluid depth.

### Visual Analogue Scale scoring

All patients will complete a VAS assessment of thoracic pain and breathlessness during their baseline assessment. After IPC insertion, beginning the following morning, patients should repeat this assessment using the documentation provided. VAS scores should then be recorded on a daily basis for the duration of trial involvement, with recordings being made each morning. If IPC drainage is due to take place that day, then the score should be noted before the drainage takes place.

### End of trial

The trial will cease recruitment once the target of 154 randomised patients has been met, or if the Trial Steering Committee feels the interim analysis after 100 patients justifies early cessation. The provisional end of trial date will therefore be 10 weeks after the randomisation of the final trial patient. At the end of each patient’s follow-up period they will be stratified as ‘alive’ or ‘dead’, and survival data collated. Further information regarding participants’ health status and survival may be obtained by accessing the NHS central register. This will require consent to be given separate to trial involvement. Those who still have an IPC *in situ* will have their care devolved to the appropriate local services.

### Patient withdrawal and loss to follow-up

Patients will have originally consented to trial follow-up procedures, including sample collection, storage and analysis where appropriate. Patients have the right to withdraw from the trial at any point. A request by a patient to withdrawal does not have to be justified and will not affect future or on-going care. In the event of withdrawal, any details available regarding the reason(s) should be recorded in the patient’s CRF. Patients may still be stratified as ‘alive’ or ‘dead’ at the end of their follow-up period, unless consent for clinical data use is withdrawn. Patients who withdraw before randomisation will not be included in the final analysis.

If a patient moves to an area outside of the trial centre catchment, every effort should be made to continue follow-up in conjunction with the new local services, or via the new GP. If this cannot be done, the patient will be recorded as ‘lost to follow-up’.

### Data collection and statistical considerations

#### Data collection

Data will be collected according to the schedule described above and in Table 2. Sites will enter data onto CRFs, which will be checked by the trial coordination centre before being entered onto an electronic database.

**Table 2:** **List of major protocol amendments**

SA01	• Clarification of randomisation target of 154 patients
	• All references to Short Form 36 Quality of Life (SF-36 QoL) questionnaire removed
	• Added an exclusion criterion: patients must have access to phone for investigator trial contact
	• Clarified sample collection and analysis
	• Clarified procedure pre-randomisation
	• Clarified that patients may also be excluded from randomisation for clinical reasons other than X-ray appearances
	• Updated summary tables and clarified pre-randomisation day nomenclature
	• Stipulated a time window in which patients must have first indwelling pleural catheter (IPC) drainage post-randomisation
	• Clarified time window in which patients may have follow-up appointments
	• Clarified wording in safety reporting section and highlighted expected minor side effects from talc
	• Updated members of the Trial Steering Committee
	• New sites added: Preston, Portsmouth and Bristol Royal Infirmary
SA02	• Change of principal investigator at Portsmouth site
SA03	• New sites added: Worcester, North Staffordshire, North Tyneside, Middlesbrough, South Manchester and Blackpool
	• Creation of letter and short trial summary for district nurses
	• Alteration to primary endpoint; changing minimal fluid volume required for pleurodesis from 20 to 50 mls
	• Change to time limit given to patients to consider patient information sheet
	• Removed requirement that trial chest X-ray must only be taken as a posterior-anterior image
	• Trial flow chart updated, allowed patients to have follow-up appointments at satellite centres
	• Allowance for patients to be approached as an inpatient but management must be as an outpatient for trial
	• Clarifications to adverse event and serious adverse event reporting procedures
SA04	• New site added: Bath
SA05	• New sites added: London, Mansfield, Stockton-on-Tees and Sheffield
	• Clarification of wording of primary endpoint, removal of duplicate secondary endpoint and addition of new secondary endpoint
	• Clarification of definition of trapped lung in trial flow chart and protocol
	• Addition of new QoL questionnaire (QLQ-C30) for all new trial participants
	• Expanded the use of pleural manometry to all centres
	• Removed the need for 0.9% saline placebo to be sourced from a particular manufacturer
	• Updated wording of how the primary outcome will be analysed
	• Updated membership of the Trial Steering Committee
SA06	• New sites added: Northampton, Ayr, Cambridge, Aintree and Hull
	• Change of inclusion criteria to require World Health Organisation (WHO) performance of two or better to be eligible, three if score will decrease to two after drainage.
	• Allow patients with previous pleurodesis as long as longer than 56 days before trial entry
	• Relax follow-up visits by allowing day 42 and 56 to be carried out over the telephone
	• Allow carers or relatives to perform chest drains after the day 28 post-randomisation visit
	• Extend recruitment period to May 2015
	• Relaxation of manometry recordings from every 100 ml to every 100 to 200 ml
	• Updated membership of the Trial Steering Committee

The following CRFs will be used during the trial: enrolment, baseline assessment, IPC insertion, day 10 assessment and randomisation, follow-up, telephone follow-up, thoracic ultrasound appearances, health resource utilisation and withdrawal.

In addition to the above, patient data will also be collected via a daily patient VAS score booklet and a daily IPC drainage volume booklet.

#### Primary endpoint

The primary endpoint is the number of patients with successful pleurodesis at 5 weeks post-randomisation. For the primary outcome measure, successful pleurodesis will be defined as the collection of less than, or equal to, 50 mls of pleural fluid on three consecutive occasions, with chest opacification on the side of the IPC less than 25%, as judged by two independent clinicians, who should be blind to treatment allocation. Information on drainage volumes will be collected in the community and during follow-up visits as described above. The X-ray for chest opacification must have been taken after the third consecutive occasion of collection of less than 50 mls of fluid, and within the 10-week follow-up period. All three occasions of collection of less than 50 mls of fluid should also occur within the 10-week follow-up period.

Patients who drain less than 50 mls of fluid on three or more occasions but who continue to have greater than 25% pleural opacification on a chest X-ray due to pleural fluid (as proven by thoracic ultrasound), will be defined as having an unsuccessful pleurodesis. If there is a clinical suspicion that the drain may be blocked then appropriate attempts to resolve this should be made prior to a definition being made.

The achievement of pleurodesis should be dated to the first drainage of less than or equal to 50 mls. Even if patients achieve the requirements for pleurodesis during the trial period, they will continue to receive fortnightly follow-up visits as originally planned until the 70-day follow-up period is complete.

Patients who die during the 10-week trial period will be assessed for whether they achieved pleurodesis success prior to death. This requires the collection of less than, or equal to, 50 mls of pleural fluid on three consecutive occasions, with chest opacification on the side of the IPC less than 25%, as judged by two independent clinicians, who should be blind to treatment allocation, with the X-ray having been taken after the third consecutive collection volume of less than 50 mls.

#### Secondary endpoints

The secondary endpoints for this trial are as follows:

1. Self-reported quality of life status, measured at 14, 28, 42, 56 and 70 days, using the EQ-5D and QLQ-C30 health questionnaires.
2. Self-reported VAS scores, measured daily from randomisation to 10 weeks post-randomisation, for thoracic pain and breathlessness.
3. Total volume of pleural fluid drained from randomisation to 10 weeks post-randomisation.
4. All-cause mortality up to 10 weeks post-randomisation.
5. Number of hospital inpatient bed-days required from randomisation to 10 weeks post-randomisation.
6. Degree of loculation of pleural fluid following talc instillation as judged by thoracic ultrasound and septation score at two-weekly intervals for the 10-week follow-up period.
7. Pleurodesis success at 10 weeks post-randomisation, as defined by consecutive fluid volume measurement.
8. Number of pleural procedures to relieve pleural fluid, excluding IPC drainage, from randomisation to up to 10 weeks.
9. Pleurodesis success at five and 10 weeks post-randomisation, as defined by total volume of fluid collected over two consecutive weeks.

As part of a secondary analysis, patients who have recorded drainages of less than or equal to a total of 250 mls of fluid over two consecutive weeks during their follow-up period (with appropriate radiological findings) will also be defined as having a successful pleurodesis. The period of two consecutive weeks may begin with any drainage which is undertaken during the post-randomisation trial period, and ends two weeks later on the same day of the week. The drainage volume recorded on this final day is included in the total volume for the two-week period. Patients must be drained no less frequently than twice per week.

In order to be defined as having a successful pleurodesis, a patient’s chest X-ray must have chest opacification on the side of the IPC of less than 25%, as judged by two independent clinicians, who should be blind to treatment allocation. The X-ray for chest opacification must have been taken after the last drainage of the two-week period, and within the overall 10-week follow-up period.

For patients who successfully drain less than or equal to 250 mls of fluid in a two-week period, the date of pleurodesis is defined as the day of the first drainage in that period. All drainages which count towards the total volume must occur within the study period.

Patients who die during the follow-up period will also be assessed for pleurodesis using measurements collected prior to death. The clinical and radiological parameters used to define successful pleurodesis by volume over time remain the same as those described above.

#### Statistical analysis plan

The primary analysis will be by the intention-to-treat principle and will include all randomised patients on whom an outcome is available [20]. All tests will be two-sided, and all analyses will be adjusted for the minimisation variables [21-24]. The primary outcome will be analysed using a time-to-event regression model, which will include mortality as a competing risk. The full statistical analysis plan for the IPC-PLUS trial will be written and ratified by the Trial Steering Committee prior to data unblinding, and will be published in a separate document.

#### Interim analysis

One interim analysis will be carried out after 100 patients are randomised in order to test for efficacy. The O’Brien-Fleming stopping rule will be used, which requires a *P* value of <0.005 for the primary endpoint in order to stop the trial early [25]. If the trial is not stopped at the interim analysis, the O’Brien-Fleming rule requires a *P* value of <0.048 at the final analysis in order to declare a statistically significant difference in the primary endpoint. The results of the interim analysis will be presented to the Independent Data Monitoring Committee, who will make a recommendation to the Trial Steering Committee as to whether the trial should stop early. This recommendation will also take into consideration other sources of evidence aside from the primary endpoint, such as secondary outcomes and safety data.

#### Subsidiary studies

In addition to the primary and secondary endpoints above, the trial will generate data to inform further sub-studies. These will relate to the trial questions which are not directly linked to the pleurodesis efficacy of talc, and the details of their analysis are beyond the scope of this protocol. Sub-studies will include the following:

1. Examining whether the measurement of pleural elastance can be used to predict lung entrapment (pleural manometry).
2. Examining whether serum levels of NT-pro BNP at baseline are related to pleurodesis success.
3. Health economic analysis: the perspective adopted in the economic analysis will be that of the English National Health Service and Social Services. As a result we will collect information on the following resource use items:
  - Intervention costs: This will entail collecting information on talc, consumables and staff time. This information will be obtained by reviewing hospital records. Should a significant between-group difference in the rates of IPC blockage and drain removal occur, these will also be included in the intervention cost analysis.
  - Follow-up costs: This will entail collecting information on patients’ use of hospital resources after randomisation. Information collected will include inpatient stays, outpatient services, use of emergency departments and ambulance costs. Information on inpatient stays will be obtained by reviewing the administrative care records in each of the participating centres.

### Trial infrastructure

The Trial Management Group is responsible for the day-to-day management of the trial. The team is responsible for all aspects of the project (such as recruitment rate, budget management, protocol adherence and so forth) and for ensuring appropriate action is taken to safeguard trial participants and the quality of the study. The Respiratory Research Unit at NBT will have responsibility for authorisation, good clinical practice (GCP) and conduct, data integrity, data checking and database integrity.

The Trial Steering Committee consists of both independent members as well as researchers working on the trial. The role of the Trial Steering Committee is to provide overall supervision of the study and monitor the progress of the trial to ensure that it is being conducted in accordance with the protocol, relevant regulations and the principles of GCP. The Sponsor will be represented at Trial Steering Committee meetings but may choose to devolve this responsibility to a named representative.

The Independent Data Monitoring Committee is independent of the trial investigators, and consists of two experienced physicians and a biostatistician. Its role is to review study safety data at regular intervals, and to provide advice to the Trial Steering Committee as to whether recruitment should continue.

### Safety reporting

Standard definitions and medical judgement will be used for the identification of adverse events, adverse reactions, the expectedness and seriousness of these events and any potential relationship to a trial intervention. Due to the population of patients involved in the IPC-PLUS trial, a high number of adverse events are to be expected. Many of these will not be related to the investigational medicinal product administration or trial-related procedures, but will be as a direct consequence of the patient’s underlying malignancy. Other events may occur as a result of a trial-related intervention, but are well-documented and regarded as normal reactions in the context of talc administration. Expected adverse events in these settings are:

1. Death due to underlying malignancy;
2. Admission due to underlying malignancy;
3. New fever after instillation of slurry or placebo (≥38°C);
4. New mild tachycardia after instillation of slurry or placebo(≥20 beats per minute over baseline);
5. New pleuritic chest pain after instillation of slurry or placebo, requiring simple analgesia (simple analgesia is defined as any medication which is not a morphine derivative or equivalent);
6. New tachypnoea after instillation of slurry or placebo (increase in respiratory rate of five or more breaths per minute over baseline) and
7. New hypoxia after instillation of slurry or placebo (to saturation of ≤92% on air, or to a level requiring additional supplemental oxygen).

If any doubt in the causality of an event exists the local investigator should inform the trial coordinator who will notify the chief investigator. Pharmaceutical companies and/or other clinicians may be asked to advise in some cases. In the case of discrepant views on causality between the local investigator and others, all parties will discuss the case. In the event that no agreement is made, the MHRA will be informed of both points of view.

## Discussion

The IPC-PLUS trial is a multicentre, randomised controlled trial which has the potential to significantly affect how patients with malignant pleural disease are treated. Although the TIME2 study published by Davies *et al*. suggested that first-line therapy for MPE might include IPCs [4], they are currently viewed by many practitioners as predominantly a second-line treatment in those patients who have not had success with a talc pleurodesis.

The combination of talc and an IPC has been used in anecdotal reports, but this is the first study to examine its utility in a robust way. Theoretically, the addition of talc to an IPC should allow for pleurodesis rates similar to that seen in bedside slurry to be maintained, but with the added benefit of outpatient management. This approach is likely to be applicable to a wide range of patients, including those with shorter life expectancies, those who want to minimise the duration of an IPC being in place or to those who have a strong preference for talc therapy but do not want to spend time in hospital. Given both the increasing use of IPCs worldwide and the availability of talc as a sclerosing agent, a positive trial outcome will likely have a global impact. A negative trial outcome, or if it is shown that the addition of talc via an IPC is detrimental to patients, would still be useful information as there remains a dearth of knowledge regarding this important population of patients.

## Trial status

IPC-PLUS gained REC approval in May 2012 and MHRA approval in June 2012. The first recruitment site gained local approval in July 2012 and opened shortly after. There are currently 16 active recruitment sites in the United Kingdom, with a further three sites in the set-up phase. Recruitment will only begin at future sites once all necessary local approvals have been granted. As of July 2014, the study has enrolled 98 patients with 60 randomisations. The trial is due to complete in May 2015.

## Abbreviations

CRF: Case report form
CT: Computed tomography
ECOG/WHO: Eastern Co-operative Oncology Group/World Health Organisation
EDTA: Ethylenediaminetetraacetic acid
EQ-5D: EuroQol 5D health questionnaire
IPC: Indwelling pleural catheter
MHRA: The Medicines and Healthcare products Regulatory Agency
MPE: Malignant pleural effusion
NBT: North Bristol NHS Trust
NHS: National Health Service
NT-proBNP: N-Terminal pro-Brain Natriuretic Peptide
PA: Posterior-Anterior
QoL: Quality of life
REC: Research Ethics Committee
VAS: Visual analogue scale.
